# Fully automatic AI segmentation of oral surgery-related tissues based on cone beam computed tomography images

**DOI:** 10.1038/s41368-024-00294-z

**Published:** 2024-05-08

**Authors:** Yu Liu, Rui Xie, Lifeng Wang, Hongpeng Liu, Chen Liu, Yimin Zhao, Shizhu Bai, Wenyong Liu

**Affiliations:** 1 Beijing Yakebot Technology Co., Ltd., Beijing, China; 2 School of Mechanical Engineering and Automation, Beihang University, Beijing, China; 3 State Key Laboratory of Oral & Maxillofacial Reconstruction and Regeneration, National Clinical Research Center for Oral Diseases, Shaanxi Key Laboratory of Stomatology, Digital Center, School of Stomatology, The Fourth Military Medical University, Xi’an, China; 4 Key Laboratory of Biomechanics and Mechanobiology of the Ministry of Education, Beijing Advanced Innovation Center for Biomedical Engineering, School of Biological Science and Medical Engineering, Beihang University, Beijing, China

**Keywords:** Biomedical engineering, Dental implants

## Abstract

Accurate segmentation of oral surgery-related tissues from cone beam computed tomography (CBCT) images can significantly accelerate treatment planning and improve surgical accuracy. In this paper, we propose a fully automated tissue segmentation system for dental implant surgery. Specifically, we propose an image preprocessing method based on data distribution histograms, which can adaptively process CBCT images with different parameters. Based on this, we use the bone segmentation network to obtain the segmentation results of alveolar bone, teeth, and maxillary sinus. We use the tooth and mandibular regions as the ROI regions of tooth segmentation and mandibular nerve tube segmentation to achieve the corresponding tasks. The tooth segmentation results can obtain the order information of the dentition. The corresponding experimental results show that our method can achieve higher segmentation accuracy and efficiency compared to existing methods. Its average Dice scores on the tooth, alveolar bone, maxillary sinus, and mandibular canal segmentation tasks were 96.5%, 95.4%, 93.6%, and 94.8%, respectively. These results demonstrate that it can accelerate the development of digital dentistry.

## Introduction

With the widespread application of cone beam computed tomography (CBCT) technology in the oral field, digital technology has gradually become the foundation of modern dental diagnosis and treatment. As a high-resolution 3D imaging technology, CBCT images can provide detailed information on the oral anatomy, and by obtaining the complex anatomy of the oral cavity with precise positioning in the images, it can guide the surgeon in planning accurate implant position to avoid damage to surrounding nerves, blood vessels, etc. To orthodontics, accurate CBCT image segmentation can effectively avoid possible bone fenestration and dehiscence issues, and are an important prerequisite for achieving accurate evaluation of orthodontic plans, which can change the current situation of relying solely on doctor experience to estimate tooth root movement; to maxillofacial surgery, an accurate jawbone model can significantly improve the efficiency and accuracy of surgery, which is a prerequisite for scheme planning automation and surgical robot precision surgery; to dental implant surgery, with the gradual application of dental implant surgery robot technology in clinical practice in recent years, its accuracy and reliability have been effectively verified, proving that it can achieve higher implantation accuracy and less surgical time than manual implantation and navigation implantation, which is the future development direction of dental implant field,^1–7^ accurate segmentation results can provide a reference for the position of the mandibular nerve canal and maxillary sinus for surgery, which is the basis for computer automatic planning of implant positions^8^ and can be used to track implant positions for accurate postoperative evaluation,^9^ at the same time, it can guide the shape of preparation holes in autologous tooth transplantation surgery. To achieve these goals, the key step is to precisely segment the oral structures of interest from CBCT images and perform 3D reconstruction.

In the field of medical imaging, the automatic segmentation of teeth, maxillary and mandibular bones, maxillary sinus, and mandibular nerve canal remains a practically and technically challenging task. Currently applied methods usually require segmentation using pre-designed manual features for tooth segmentation,^10–12^ such as level sets,^13,14^ template fitting,^15^ or manual adjustment after merely preforming threshold segmentation.^16^ However, due to the complexity of the dental occlusal surface, the flexibility of tooth topology changes, the low contrast between the root and the alveolar bone, and the uncertainty of third molar eruption make it difficult for existing segmentation methods to obtain accurate segmentation results. In addition, the efficiency of the existing tooth image segmentation methods is too low, which leads to a significant reduction in the efficiency of the dental implant surgery robot, and the professional dentist needs to spend more time on surgical planning and image processing compared to traditional freehand implants, and cannot focus on the surgical plan design.

And with the continuous development of deep learning methods, such as deep learning based on convolutional neural networks (CNNs), which have shown great robustness and accuracy in the field of medical images,^17–26^ a series of studies related to the application of deep learning methods for the segmentation of tooth, bone, and mandibular neural tube structures have emerged.^27–31^ Among these studies, the tooth instance segmentation task is one of the most important and complex tasks, existing methods typically require designing complex tooth morphology representations and attaching tooth prior knowledge. Cui et al.^32^ first proposed the first network for tooth CBCT segmentation task in 2019: ToothNet. This method first extracts tooth edges from the input CBCT image, and then sends the detected edge map and the original CBCT image to the region proposal network to obtain segmentation results. Lee et al.^33^ and Gerhardt et al.^34^ proposed a method of first obtaining the position of a single tooth from the original CBCT image through object detection and other methods for segmentation, dividing the segmentation task into two stages and reducing the complexity of the network. To obtain better segmentation results, Cui et al.^27^ proposed to learn the tooth centroids and skeletons for identifying each tooth’s rough position and topological structures, respectively. Chung et al.^31^ proposed to first realign the CBCT image according to the maximum intensities projection. Liu et al.’s^35^ method requires first automatically registering the intraoral scan model obtained by an intraoral scanner or scanning traditional oral impressions with the CBCT image, and then segmenting the CBCT image based on the segmentation results of the intraoral scan model. Second, current methods are based on CNN networks, which have major problems in modeling long-range information because of the inherent properties of convolutional kernels^24^ and obtaining accurate the adjacent relationship between teeth and missing teeth. In addition, due to significant differences in grayscale range, contrast, and field of view between different CBCT images, existing methods have not optimized for this.^36^

Summarizing the current methods, four shortcomings can be found as follows:

1. Tooth segmentation results of existing methods do not mark the teeth according to the FDI Two-Digit Notation,^37^ so that the missing tooth information cannot be accurately detected and represented. We reproduced the method of Cui et al.^27^ which is the state-of-the-art in this field, the result is shown in Fig. 1. It cannot achieve the same classification results for teeth in the same position, which is mapped in the figure as different colors of teeth in the same position. The method proposed by Liu et al.^35^ requires first segmenting the oral scan model and registering it with the CBCT image to obtain segmentation results. This limits the application of these methods in the analysis and diagnosis of dental defects, since information on missing teeth is essential for the correct localization and assessment of the patient’s oral health status.

**Fig. 1:**
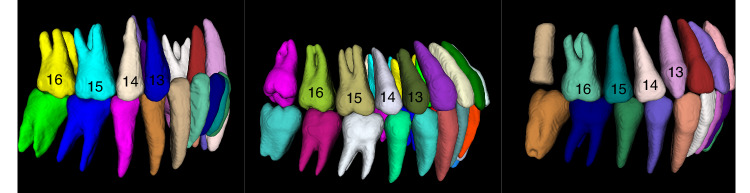
Segmentation results of existing algorithms for tooth roots (upper and lower right regions, as can be seen, the same teeth 13, 14, 15, 16 are labeled as different teeth)
2. Unlike conventional CT, different CBCT equipment manufacturers use different imaging devices and interpolation methods, and also dynamically select imaging parameters according to the patient’s condition when in use, so CBCT images will have different grayscale distribution and contrast, which makes it difficult to apply simple image preprocessing methods to all CBCT images; and in the tooth instance segmentation task, most of the original images are soft tissue regions that are not useful for segmentation, resulting in an unbalanced distribution of data, which is not addressed by the current methods. The actual CBCT images are shown in Fig. 2, and it can be seen that there are large differences in image contrast, field of view, etc.

**Fig. 2:**
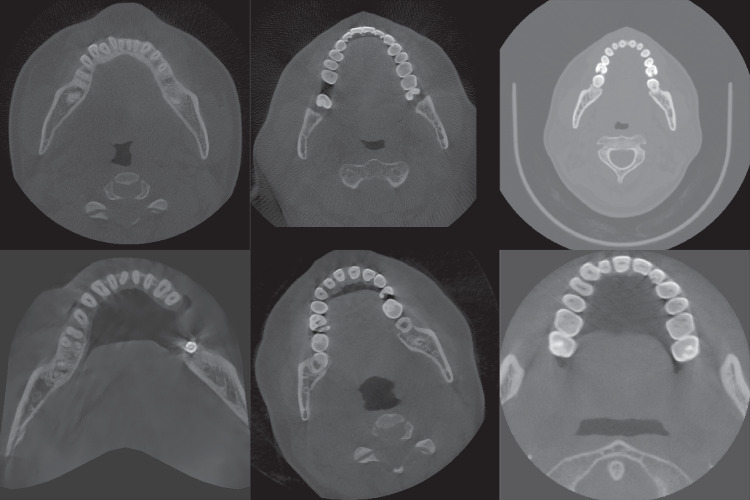
Slices of CBCT data from different sources
3. Existing methods are too complex and often require multiple steps to obtain good segmentation results. These methods may require the use of multiple preprocessing techniques, feature extraction methods, classifiers or models, and post-processing steps to complete the segmentation of teeth. This complexity leads to less computationally efficient methods, each step may introduce errors or mistakes, and each step in the whole process requires careful tuning and validation, which increases the difficulty of method development and application.
4. Current methods do not perform all these segmentation tasks completely automatically in an end-to-end manner, as they usually focus on a single task, such as tooth segmentation or alveolar bone segmentation on predefined region of interest (ROI), with little research on the segmentation of the mandibular canal from the maxillary sinus.

These aforementioned drawbacks limit the accuracy, generalizability, and efficiency of these methods for oral structure segmentation and diagnostic tasks.

In this paper, we propose a deep learning-based fully automated segmentation system aimed at the precise delineation of tissues relevant to dental implantation. Specifically, we propose a data distribution histogram-based image preprocessing method by statistically analyzing the data distribution histograms of different brands of CBCT images. Based on this, we use a skeletal segmentation network to obtain maxillary and mandibular bone, tooth, and maxillary sinus segmentation results, and use the tooth and mandibular bone segmentation results as the ROI for subsequent tooth instance segmentation and mandibular neural tube segmentation, respectively. For the tooth segmentation, an attention-based deep learning network is proposed to obtain accurate tooth instance segmentation results, and the segmentation results can be labeled according to the FDI Two-Digit Notation^37^ to obtain information about the patient’s tooth missed sit; for the mandibular canal segmentation, we design a multilayer hierarchical feature extraction neural network to perform this task. The corresponding experimental results show that our method can obtain more accurate segmentation results and higher efficiency than the current methods, and the proposed data preprocessing method can effectively improve the segmentation accuracy.

## Results

**Fig. 3:**
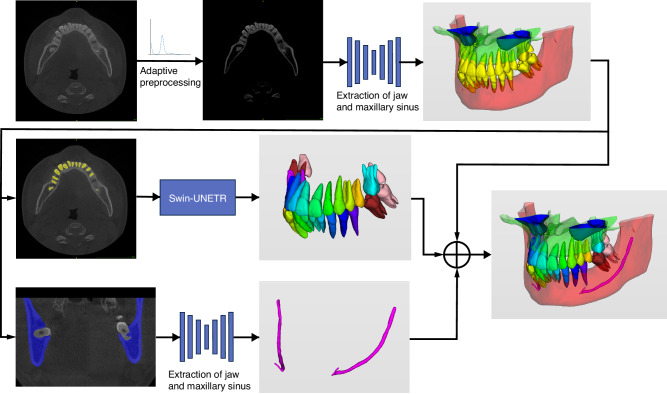
Overview of our proposed artificial intelligence system for segmenting individual teeth, maxillary and mandibular bones, maxillary sinus, and mandibular neural tube from CBCT images (The input to the system is a 3D CBCT scan of the patient; a uniform image is first obtained using a CBCT image adaptive preprocessing algorithm, then processed by a bone extraction, neural tube extraction and tooth instance segmentation network, and finally a mask containing all structures to be segmented is output)

An overview of the proposed method applied to the segmentation of oral CBCT images is illustrated in Fig. 3, it consists of three parts. First, the image preprocessing method proposed in this paper is applied and the preprocessed images are used for subsequent training. In the deep learning processing stage, a skeletal segmentation network is used to segment the preprocessed images to obtain four types of segmentation results: teeth, maxillary bone, mandibular bone, and maxillary sinus. *Then, the tooth segmentation results and mandibular bone segmentation results are used as ROIs* regions for the subsequent tasks, and the subsequent tooth instance segmentation results are combined with neural tube segmentation results and bone segmentation results to obtain the final oral CBCT image segmentation results.

**Fig. 4:**
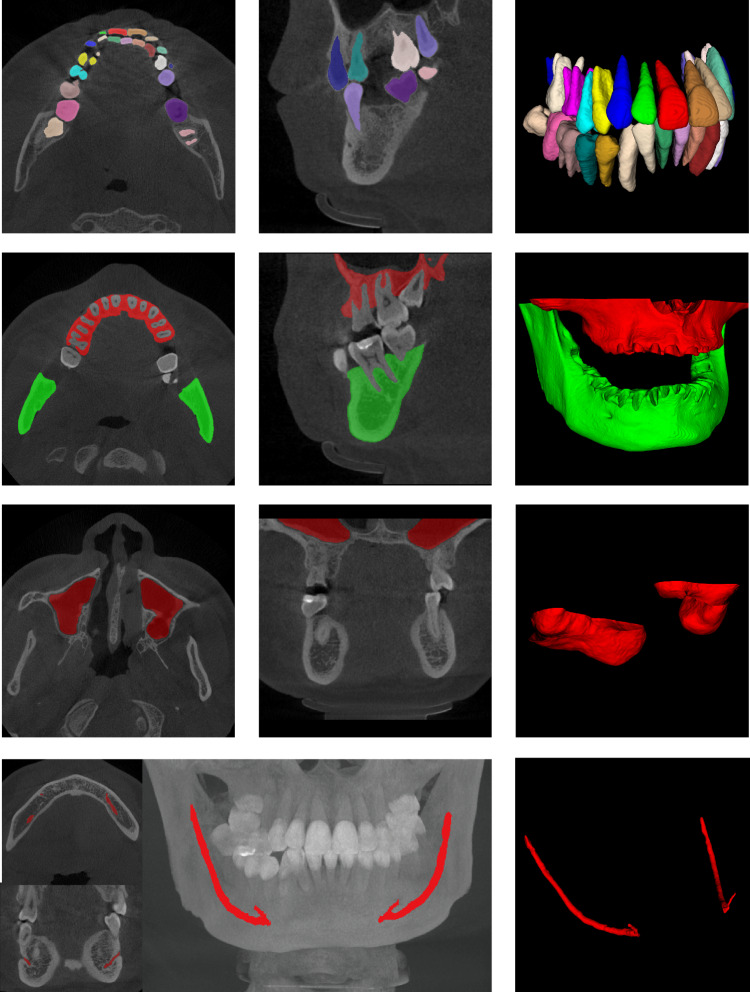
Results of tooth, maxillary and mandibular bone, maxillary sinus, and mandibular nerve canal segmentation

Table 1 shows the segmentation accuracy achieved by our proposed method on the validation dataset, containing tooth segmentation, maxillary and mandibular segmentation, maxillary sinus segmentation with mandibular neural tube segmentation. As can be seen, on the test dataset, our AI system was able to obtain an average Dice score of 96.5%, an average mean Intersection over Union (mIoU) of 88.4%, an average Hausdorff distance (HD) of 1.62 mm, and an average surface distance (ASD) error of 0.12 mm in the segmentation of teeth task; while it was able to achieve 92.4%, 98.3%, 93.6%, and 94.8% in the maxillary, mandible, maxillary sinus, and mandibular neural tube segmentation tasks, respectively. Figure 4 shows the segmentation results for the teeth, maxillary and mandibular bones, maxillary sinus, and mandibular canal, respectively.

**Table 1:** Segmentation accuracy of teeth, maxillary and mandibular bones, maxillary sinus, and mandibular nerve canal on the dataset

Segment classes	Dice/%	mIoU/%	HD/mm	ASD/mm
Tooth	96.5 ± 0.8	88.4 ± 0.6	1.62 ± 0.12	0.12 ± 0.12
Maxillary bone	92.4 ± 3.1	79.5 ± 1.0	4.25 ± 0.61	0.49 ± 0.76
Mandible bone	98.3 ± 1.8	93.4 ± 0.8	0.97 ± 1.58	0.11 ± 0.54
Mandibular canal	94.8 ± 0.9	82.0 ± 2.1	1.53 ± 0.22	0.35 ± 0.98
Maxillary sinus	93.6 ± 1.7	84.8 ± 0.7	1.12 ± 0.85	0.28 ± 0.23
Average	95.1 ± 1.5	85.6 ± 1.1	1.90 ± 0.68	0.27 ± 0.53

We compared our proposed method with deep learning methods from previous years, including Hi-MoToothSeg,^38^ nnUNet,^39^ ToothNet,^32^ RELU-Net,^34^ and DenseASPP-UNet,^40^ all applying common data preprocessing methods to validate the effectiveness of our proposed method. The results are shown in Table 2, and it can be seen that our method achieves higher accuracy in all four metrics compared to current methods. Meanwhile, we conducted ablation experiments to verify the effectiveness of our proposed pretreatment method, and the results are shown in Table 3. It can be seen that our adaptive preprocessing method achieves a large improvement in all four metrics compared to the generic preprocessing method.

**Table 2:** Experimental results of quantitative comparison with existing advanced methods in terms of segmentation and detection accuracy

Methods	Dice/%	mIoU/%	HD/mm	ASD/mm
Hi-MoToothSeg	93.1 ± 0.8	82.5 ± 1.8	1.63 ± 0.75	0.28 ± 0.14
nnUNet	85.3 ± 2.5	75.1 ± 2.0	5.04 ± 2.48	0.51 ± 0.31
ToothNet	91.7 ± 1.3	76.2 ± 0.7	2.85 ± 1.11	0.49 ± 0.08
RELU-Net	92.9 ± 1.0	85.7 ± 0.9	1.52 ± 0.42	0.24 ± 0.11
DenseASPP-UNet	92.5 ± 1.4	79.4 ± 1.2	2.34 ± 0.76	0.31 ± 0.21
Ours	**94.3** ± **1.0**	**86.3** ± **1.1**	**1.43** ± **0.52**	**0.18** ± **0.04**

Bold text represents the highest value in its column

**Table 3:** Analysis of the effect of choosing different relationships between *d* and *σ* on segmentation accuracy during image preprocessing

Items	Dice/%	mIoU/%	HD/mm	ASD/mm
Normal preprocess	94.3 ± 1.0	86.3 ± 1.1	1.73 ± 0.52	0.18 ± 0.04
*d* = 0 × *σ*	94.9 ± 1.2	86.9 ± 1.0	1.68 ± 0.87	0.18 ± 0.21
*d* = 1 × *σ*	95.8 ± 0.7	87.5 ± 0.6	1.63 ± 0.38	0.15 ± 0.34
*d* = 2 × *σ*	96.2 ± 0.6	**88.9** ± **0.6**	1.65 ± 0.18	0.13 ± 0.08
*d* = 3 × *σ*	**96.5** ± **0.8**	88.4 ± 0.6	**1.62** ± **0.12**	**0.12** ± **0.12**
*d* = 4 × *σ*	96.5 ± 1.1	88.7 ± 1.2	1.64 ± 0.21	0.12 ± 0.68
*d* = 5 × *σ*	95.9 ± 0.7	86.8 ± 0.9	1.69 ± 0.32	0.14 ± 0.10
*d* = 6 × *σ*	95.2 ± 1.8	86.1 ± 1.6	1.70 ± 0.31	0.16 ± 0.49

Bold text represents the highest value in its column

**Fig. 5:**
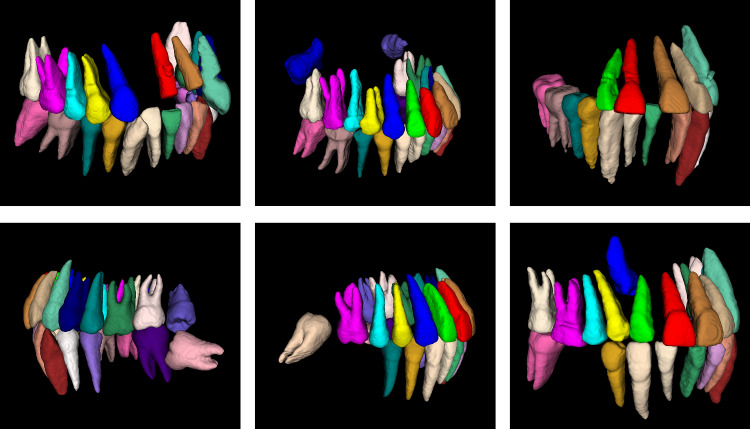
Segmentation results for different CBCT data (teeth with the same number are shown as the same color when displayed using the same color mapping relationship)

Moreover, our method was able to obtain accurate relative tooth positions, and teeth in the same position were able to obtain the same markers, thus enabling us to assess the segmentation accuracy for each specific tooth, and the segmentation results are shown in Fig. 5. We chose the Dice coefficient as the evaluation index and evaluated the segmentation accuracy of maxillary and mandibular central incisors (T1), lateral incisors (T2), canine/acute teeth (T3), first premolars (T4), second premolars (T5), first molars (T6), second molars (T7), and third molars (T8), respectively, and the results are shown in Table 4.

**Table 4:** Segmentation accuracy of Dice for teeth in different positions

Methods	T1	T2	T3	T4	T5	T6	T7	T8
Ours	97.1 ± 1.2	96.8 ± 0.5	97.0 ± 0.2	93.7 ± 1.4	94.1 ± 1.7	96.5 ± 0.8	92.3 ± 2.5	93.1 ± 2.1

In summary, our method is able to identify the relative positions of teeth while obtaining higher accuracy, obtain segmentation results marked according to the FDI Two-Digit Notation, and achieve higher segmentation efficiency with better clinical application prospects.

Taking the tooth segmentation task as an example, we validated the generalizability of the proposed method for different CBCT data on the independent validation dataset constructed above, and the validation results are shown in Table 5. It can be seen that our method achieves good performance on CBCT data using different image protocols, where it is able to obtain the best performance on the LargeV dataset, reaching an average Dice score of 96.9%.

**Table 5:** Segmentation accuracy performance of the proposed tooth instance segmentation method on CBCT images from different sources

Manufacturer	Manufacturer’s model name	Dice/%	mIoU/%	HD/mm	ASD/mm
Carestream Health	CS 9300, CS 9301	95.9 ± 1.2	89.1 ± 1.0	1.45 ± 0.23	0.17 ± 0.25
Imaging Sciences International	9–17	95.3 ± 1.2	88.6 ± 0.2	1.42 ± 0.21	0.18 ± 0.11
J.Morita.Mfg.Corp.		96.2 ± 0.8	90.3 ± 0.9	1.15 ± 0.31	**0.09** ± **0.08**
LargeV	HighRes3D, SMART3D	**96.9** ± **0.3**	**91.2** ± **0.8**	1.22 ± 0.25	0.09 ± 0.10
NewTom	NTVGiMK4, NTVGiEVO, NT5G	93.8 ± 1.5	86.7 ± 0.6	**1.10** ± **0.25**	0.16 ± 0.13
NNT	NTVGiEVO	96.1 ± 1.2	89.8 ± 0.9	1.22 ± 0.42	0.15 ± 0.32
PaloDEx Group Oy	ORTHOPANTOMOGRAPH OP 3D	94.8 ± 0.9	87.9 ± 1.1	1.81 ± 0.21	0.18 ± 0.23
RAY Co., Ltd.	RAYSCAN N Alpha Plus	95.9 ± 1.1	89.4 ± 0.7	1.84 ± 0.25	0.15 ± 0.14
Sirona	ORTHOPHOS SL	94.1 ± 2.1	86.8 ± 1.1	2.72 ± 0.62	0.42 ± 0.52
Vatech Company Limited	PHT-35LHS	93.7 ± 3.8	85.5 ± 2.0	2.12 ± 0.40	0.32 ± 0.24
YOFO	Pirox-R	96.1 ± 1.2	90.1 ± 0.8	1.21 ± 0.35	0.16 ± 0.09

Bold text represents the highest value in its column

Finally, we experimentally verify the efficiency and accuracy of the proposed fully automated end-to-end segmentation method compared to the traditional expert manual outlining method. The experimental results are shown in Table 6, where we can see that our method can achieve hundreds of times higher efficiency and the same segmentation accuracy as the expert.

**Table 6:** Quantitative comparison of segmentation accuracy and segmentation time between our AI system and a dental expert (three CBCT images randomly selected from the dataset)

Items	AI	Expert	AI-assist + hand-tuning
Time cost/min	**1.52**	240	5.6
Dice/%	**95.7**	92.6	

Bold text represents the highest value in its column

## Discussion

In this study, we present a deep learning-based method for segmenting teeth, maxillary and mandibular bones, maxillary sinus, and mandibular nerve canal in oral CBCT images. Our method has several distinguishing features that make it different from current methods.

First, we achieve an end-to-end segmentation of all the necessary structures required for dental implant surgery planning and robotic navigation of the dental implant. This feature is critical because it allows for a streamlined and efficient workflow in treatment planning without the need for manual annotation or multiple individual segmentation steps. Experiment results show that our approach can significantly improve efficiency and accuracy compared to experts manually outlining ROI using existing software.

Second, we developed an adaptive algorithm that can efficiently handle the variations of CBCT images acquired from different manufacturers. This adaptation is critical because CBCT images may exhibit variations in image quality, resolution, noise, and contrast due to differences in acquisition protocols and equipment, and current methods are not targeted for CBCT images, making them less generalizable and requiring increasing data volumes to improve the applicability of the method. By adapting to these variations, our method is able to achieve the same or even better segmentation accuracy on a smaller training dataset. Experimental results show that our method exhibits robust performance on data from multiple CBCT manufacturers, improving the average Dice score by 3.6% in the most dominant tooth instance segmentation task compared to the best available method, and by applying the proposed adaptive preprocessing method, it also achieves an average Dice score improvement of 2.8% compared to itself.

An important contribution of our study is the ability of the proposed tooth instance segmentation method to obtain tooth classification results based on the FDI Two-Digit Notation. This feature enables automatic identification of missing teeth, which is an important aspect of dental analysis and treatment planning. Current methods often lack the ability to accurately capture information about missing teeth^32,38,40^ or require the use of oral scan models^35^ which can make the practical application of the algorithm difficult and make it impossible to accurately locate and select specific teeth during software interaction. The ability to accurately capture information about missing teeth makes our method more conducive to comprehensive dental evaluation.

To evaluate the performance of our method, we conducted extensive experiments and compared it with existing techniques. The results demonstrate the effectiveness and accuracy of our method in segmenting teeth, maxillary and mandibular bones, maxillary sinus, and mandibular nerve canal, with an overall average Dice score of 95.1% and the ability to achieve state-of-the-art on the most important tooth segmentation tasks, indicating the robustness and generalizability of our method in capturing anatomical structures of interest.

In addition, we evaluated the impact of the adaptive algorithm on different CBCT images obtained from different manufacturers. The results show the superiority of our adaptive approach compared to conventional methods, as it consistently achieves high segmentation accuracy regardless of the imaging characteristics of the CBCT system.

Although our research results promise competitive results, there are some limitations that need to be acknowledged. First, the evaluation is conducted on a specific dataset, which does not include severe metal artifacts or image blurring caused by patient movement issues. Therefore, further exploration should be conducted on the generalizability of this method to a wider population and various clinical scenarios. Second, although our adaptive algorithm showed effective performance, there is still room for improvement, such as severe metal artifacts in images,^41^ deep learning-based CBCT image grayscale value processing,^42,43^ and the combination of segmentation results with vitro experiments.^44^

## Conclusion

In this paper, we present a comprehensive study on the segmentation of dental implant structures of interest in oral CBCT images using deep learning techniques. Our proposed method provides several new features and demonstrates a significant improvement over current methods.

We have successfully developed an end-to-end segmentation method that contains the necessary information required for dental implant surgical planning. Unlike current methods that require manual outlining or segmentation in multiple steps, our method simplifies the image segmentation and surgical planning process and is the basis for fully automated planning of dental implant surgery, saving the clinician valuable time and effort.

In addition, we propose an adaptive algorithm capable of handling the variations inherent in CBCT images obtained from different manufacturers. This adaptability ensures the consistency and robustness of deep learning methods on multiple CBCT images, overcoming the challenges posed by differences in image quality, resolution, noise, and contrast.

A notable contribution of our work is the direct extraction of tooth segmentation results based on the FDI Two-Digit Notation. This enables the automatic identification of missing teeth, a key aspect of dental analysis and treatment planning. Unlike current methods, our approach successfully captures information about missing teeth, improving the comprehensiveness of dental assessments.

Our extensive experimental evaluation and comparison with existing techniques demonstrated the superiority and accuracy of our method in segmenting the teeth, maxillary and mandibular bones, maxillary sinus, and mandibular nerve canal. The results confirm the robustness of our method in capturing the anatomical structures of interest, even at different tooth positions.

Although our study yielded remarkable results, it is important to acknowledge its limitations. Our evaluation was performed on a specific dataset and further investigation is necessary to assess the generalizability of our approach to different populations and clinical scenarios, and to further validate the efficiency and accuracy of the method through broader integration with clinical practice. In addition, there is potential for further improvement in handling extreme variations in CBCT image features, which should be addressed in future studies.

In conclusion, our study introduces a new deep learning-based method to segment structures in oral CBCT images. The end-to-end segmentation capability of all structures required for dental implant surgery, the segmentation capability based on the FDI Two-Digit Notation, the adaptability to different CBCT images, and the ability to extract information about missing teeth offer significant advantages over current methods. The experimental results demonstrate the effectiveness and accuracy of our approach and the ability to significantly reduce the preoperative planning time consumed by the surgeon when performing surgery with the dental implant surgery robot. Further research should focus on validating our method on larger and diverse datasets and addressing the identified limitations to enhance its robustness and applicability in clinical practice.

## Methods

### CBCT image adaptive preprocessing method

CBCT images use a grayscale density value scale, which is similar to the HU value of ordinary CT. However, the difference is that the HU value is fixed, usually set to 0,^45^ and the range of grayscale values and contrast of CBCT images can vary depending on the interpolation method chosen by the equipment manufacturer, the imaging equipment, the parameters chosen during scanning and the field of view, etc. Moreover, the voxels containing teeth in CBCT images only account for about 1%–3% of the whole image, which causes unbalanced distribution of categories and slows down the training speed and accuracy of the network. The existing methods do not provide targeted processing for the above characteristics of CBCT images, only using general image preprocessing methods, which delete the first 0.5% and last 99.5% data of the image, and then perform regularization.

**Fig. 6:**
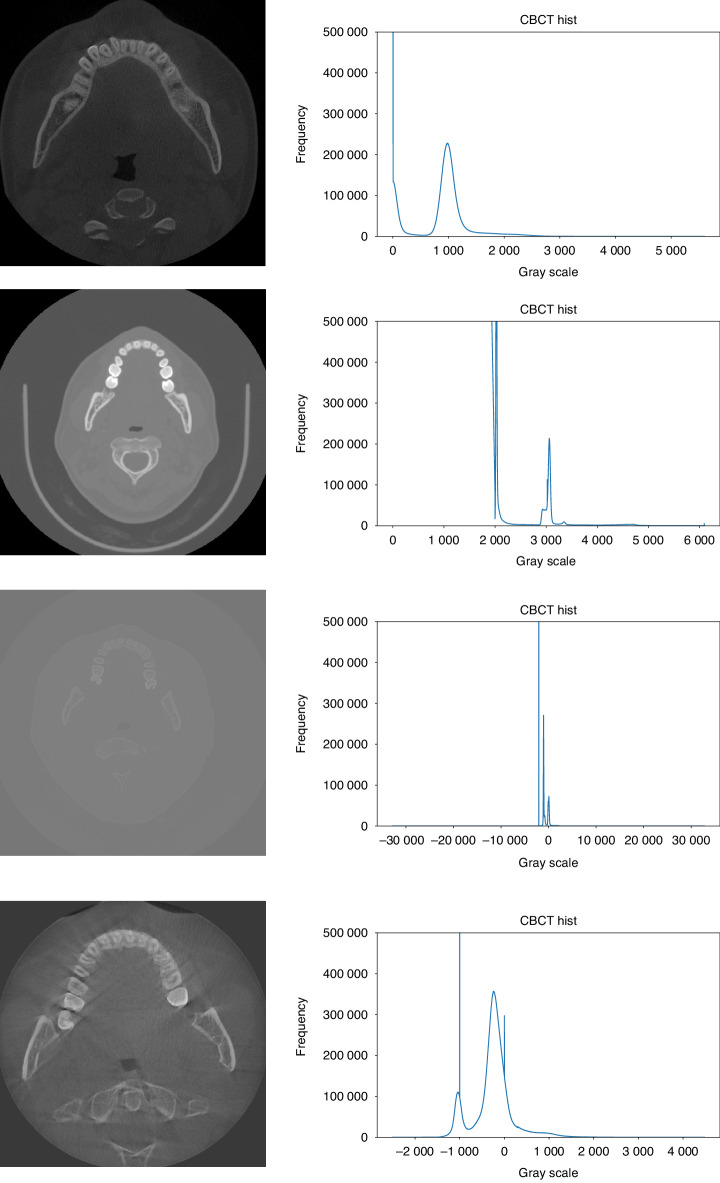
CBCT image slices with different field of view, gray value range and contrast ratio and corresponding gray histograms

After performing CT value distribution statistics on the existing dataset, we plotted the histogram of grayscale value distribution for each data, and Fig. 6a–d shows several typical distributions of grayscale values of CBCT images in the dataset. It can be seen that each image usually contains one or two spikes and one peak. Based on the original image, it can be seen that the spikes represent the air in the image, while the peak represents the grayscale value of the soft tissue. The goal of image preprocessing is to delete data with a grayscale value smaller than the bone tissue and then perform normalizing processing.

Looking at the statistical histogram, it can be seen that the CBCT images vary in their grayscale range and that one or two spikes appear because the voxels appearing outside the field of view and the voxels representing air have the same grayscale value, respectively; on the other hand, since most of the images are of soft tissues, a peak appears, whereas there is no significant undulation of bones, teeth, and artificial restoration. Therefore, the average grayscale value *x*_s_ for soft tissue can be obtained by calculating the midpoint of the soft tissue peak in the histogram, while the truncated gray value for bone is:

1 $${x}_{\rm{b}}={x}_{\rm{s}}+d$$

where *d* is the difference between the soft tissue grayscale value and the bone grayscale value.

To obtain the values of *x*_s_ and *d*, we consider the histogram of the grayscale distribution as a digital signal and introduce frequency domain processing methods for signal processing, and finally obtain the requested values. The detailed method is as follows.

First, we consider the calculated histogram of the gray distribution as a signal curve representing the frequency of captured gray values for further analysis. To improve the curve quality, a median filter is applied to remove spikes or noise artifacts.

Next, the midpoint of the soft tissue peak on the filtered signal curve is determined, and this point is the *x*_s_ to be calculated. To eliminate the impact of curve fluctuations on peak calculation, it is necessary to set a minimum threshold for the width and height of the target peak. In this experiment, the width was set to 5, and the height was set to 0.1% of the total number of pixels in the image through statistical analysis of the dataset used.

Subsequently, define the ROI around the peak by selecting a width of 200 units on both sides of the midpoint of the peak. We assume that the peak in this area obeys the Gaussian distribution, and select the Gaussian distribution density function to model the ROI. The functional equation is:

2 $$f\left(x\right)=\frac{k}{\sqrt{2\pi }\sigma }{e}^{\frac{-{\left(x-\mu \right)}^{2}}{2{\sigma }^{2}}}$$

where *μ* is the midpoint of the wave, *σ* is the standard deviation, and *k* is the amplitude.

Finally, based on the fitted Gaussian distribution function, the relationship between the soft tissue and bone gray value disparity *d* and *σ* to be calculated is obtained experimentally.

**Fig. 7:**
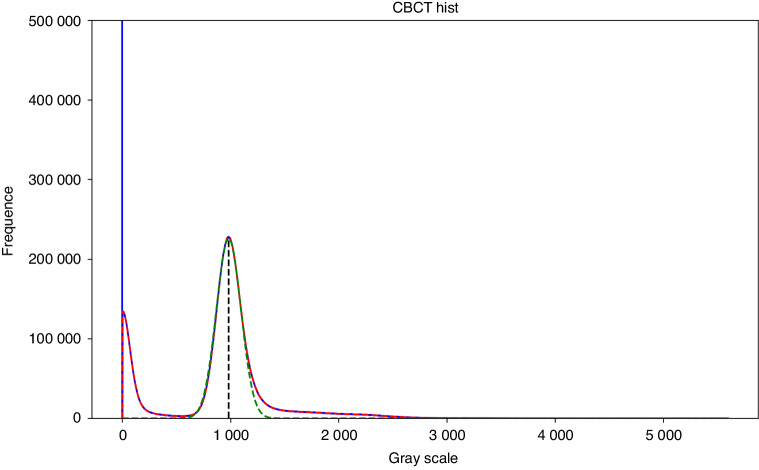
Histogram of grayscale distribution of CBCT images with preprocessing results

Figure 7 illustrates the above processing flow, where the blue curve is the original histogram, the red curve is the curve after median filtering, the green curve is the fitted Gaussian distribution probability density function, and the black line segment is the wave peak midpoint.

**Fig. 8:**
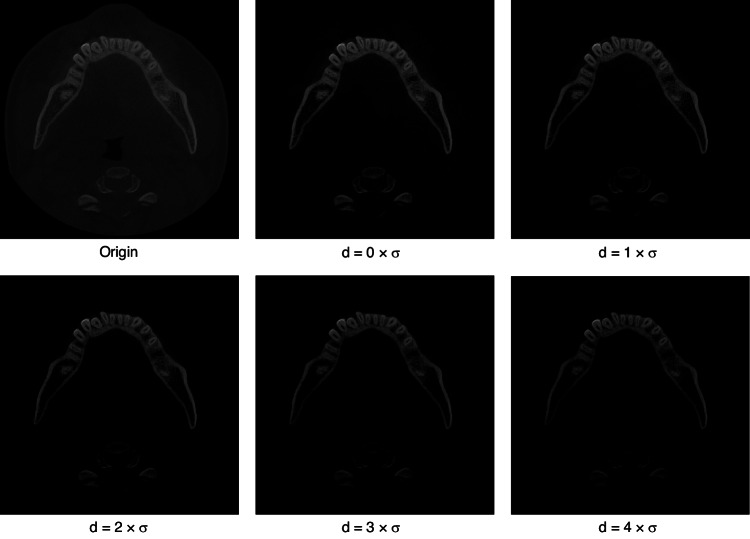
Images when different preprocessing parameters are selected separately

Figure 8 shows the CBCT image slices after the application of the above preprocessing method, in relation to the selected *d* and *σ*.

### Network structure

For the two stages of skeletal segmentation and neural tube segmentation, we selected the 3D-UNet network^39,46^ as the network backbone. The Swin-UNETR network was selected for the tooth instance segmentation stage to achieve this task. Due to the limitations of the size of the convolutional kernel and the number of filter channels in CNNs, the receptive field is limited, making it difficult to capture and store long-range dependency information.^25^ In order to capture long-range dependencies, it is usually only possible to increase the size of the convolutional kernel or the number of channels, which can lead to gradient vanishing or dimensional disasters. In terms of the problem to be solved in this paper, the above problems will make it difficult to obtain the relationship between two teeth that are far away when performing tooth instance segmentation, which leads to low segmentation accuracy. The existing CNN-based tooth instance segmentation methods require other feature information as input, such as pre-obtained tooth centroids, tooth positions obtained using oral scan models, etc.

The transformer structure in the NLP domain is inherently capable of capturing long-range dependency information, thus achieving significant success in the field of computer vision in recent years.^8,17,22,47–49^ In the field of tooth instance segmentation, the localization of teeth according to the FDI Two-Digit Notation and the identification of missing teeth depend mainly on their adjacent teeth and the overall dentition information, which makes it necessary that the applied deep learning network also has the ability to model between pixels at long distances and requires the network to be able to learn local features of the image at the same time due to the close grayscale values and blurred boundaries of the teeth and jaws in the root portion of the tooth. Therefore, we chose the Swin-UNETR^25^ network, which has demonstrated excellent performance in semantic segmentation of brain tumors, as the segmentation network. This method can simultaneously model the relationships between long-distance pixels and extract local information, which is crucial for predicting tooth position.

The Swin-UNETR model consists of the following components:

Swin-Transformer: as a feature extractor, used to extract meaningful feature representations from the input image. It is based on the Swin-Transformer architecture^50^ and uses non-overlapping sliding windows at multiple levels for feature extraction through a self-attentive mechanism and a fully connected network. This feature extraction method takes full account of the global contextual information and local detail information of the image, which helps to improve the semantic segmentation performance.

Encoder part: it includes several UNetR base modules^51^ for gradually decreasing feature dimensionality. The encoder extracts a more abstract and semantically rich feature representation from the output features of the Swin-Transformer through multiple layers of convolution and normalization operations. Each encoder block contains convolution operations and residual concatenation, which help to preserve important feature information and mitigate the gradient disappearance problem.

Decoder part: includes multiple UNetR upsampling blocks for gradually restoring the feature dimension to its original size. The decoder recovers the lost detail information by fusing the encoder features with higher resolution features through upsampling operations and jump connections. This improves the accuracy and precision of the segmentation results.

Output layer: used to generate the final segmentation prediction results. The output layer maps the decoder features to the probability distribution of the target class through a series of convolution and normalization operations. The final output results can be used for semantic segmentation at the pixel level.

The network structure of the Swin-UNETR model takes full advantage of the Swin-Transformer to enable fine-grained segmentation tasks with the encoder–decoder design of the UNet structure while maintaining the global perception capability. In this task, the selected hyperparameters are shown in Table 7.

**Table 7:** Network hyperparameters selection

Embed dimension	Feature size	Number of blocks	Window size	Number of heads	Parameters	FLOPS
768	48	[2,2,2,2]	[7,7,7]	[3,6,12,24]	62.19M	394.84G

### Loss function

We weighted the results of the CE loss and Dice loss^26^ calculations together as the loss function at training.

3 $${\rm{Loss}}={w}_{\rm{CE}}{\rm{Loss}}_{\rm{CE}}+{w}_{\rm{Dice}}{\rm{Loss}}_{\rm{Dice}}$$

where *w*_CE_ and *w*_Dice_ are the weights of CE loss and Dice loss, respectively, which are both set to 1 in this experiment.

4 $${\rm{Loss}}_{\rm{CE}}=-\frac{1}{C}\mathop{\sum }\limits_{j=1}^{C}\mathop{\sum }\limits_{i=1}^{N}{w}_{j}{G}_{i,j}\log \left({P}_{i,j}\right)$$

5 $${\rm{Loss}}_{\rm{Dice}}=1-\frac{2}{C}\mathop{\sum }\limits_{j=1}^{C}\frac{{\sum }_{i=1}^{N}{P}_{i,j}{G}_{i,j}}{{\sum }_{i=1}^{N}{P}_{i,j}^{2}+{\sum }_{i=1}^{N}{G}_{i,j}^{2}}$$

where *C* is the number of categories; *N* is the total number of voxels; *w*_*j*_ is the weight of category *j*; *P*_*i,j*_ is the probability that the *i*-th voxel belongs to category *j* as output from the model; *G*_*i,j*_ is the probability that the *i*-th voxel belongs to category *j* after encoding the ground truth with one-hot code.

### Dataset

In this study, we collected a total of 451 CBCT data with entire dental arch from 10 different medical institutions and publicly available datasets, including 11 CBCT manufacturers and 13 imaging modalities, to evaluate the accuracy of the proposed method, excluding CBCT blurring caused by patient motion or insufficient imaging parameters during the imaging process. The detailed imaging protocols (i.e., image resolution, manufacturer, manufacturer’s model name, and radiation dose information for tube current and tube voltage) and patient age–sex distribution of the data are shown in Table 8. At the same time, we also collected a total of 55 CBCT data from the 10 medical institutions mentioned above, including 11 CBCT manufacturers, to verify the generalizability of the proposed method.

**Table 8:** Description and characteristics of the CBCT datasets from different medical institutions (only voxel size is available in the public dataset)

Manufacturer	Manufacturer’s model name	Sex (Female/Male)	Tube voltage/kVp	Tube current/mA	Spacing/mm	Average age/years	CBCT number (cases)
Carestream Health	CS 9300, CS 9301		90	10	0.18	40.1	15
Imaging Sciences International	9–17	10F/4M	120	5	0.2	44.1	14
J.Morita.Mfg.Corp.		2F/2M	89	7	0.25	25.3	4
LargeV	HighRes3D, SMART3D	60F/52M	100	4	0.25	44.5	112
NewTom	NTVGiMK4, NTVGiEVO, NT5G	22F/13M	110	1, 2, 3, 4, 5, 7, 9, 10	0.3, 0.25	42.1	35
NNT	NTVGiEVO	7M	110	3, 7, 8, 9, 10, 11, 14	0.3	31.2	7
PaloDEx Group Oy	ORTHOPANTOMOGRAPH OP 3D	5M	95	3, 8	0.25	44.2	7
RAY Co., Ltd.	RAYSCAN N Alpha Plus	1F/2M	90	10	1		3
Sirona	ORTHOPHOS SL		85	10	0.22	46.7	10
Vatech Company Limited	PHT-35LHS	1F/3M	94	8	0.2	47.8	4
YOFO	Pirox-R	29F/23M	90	8	0.25	40.1	52
					0.4		91
					0.4		97

CBCT images were labeled under the guidance of professional dentists to obtain the gold standard. The dataset was randomly divided into three categories: training set, validation set, and test set, while all personal information of patients is removed.

The physical resolution of the CBCT images we collected was distributed from 0.18 to 1.0 mm. Considering the clinical application and data processing efficiency, it was, therefore, necessary to first resample the data according to the physical resolution of 0.4 mm. The resampled CBCT data were first preprocessed by the preprocessing method proposed above, and the retained grayscale values ranged from the grayscale values of the bones obtained by preprocessing to 99.5% of the overall, and then normalized to obtain a standard image with a mean of 0 and a standard deviation of 1. During the training process, data of size 96 × 160 × 160 were randomly cropped from the 3D images as training data. In order to improve the generalizability of the model, we also apply random mirror flip and random add mask methods to enhance the data, where random mirror flip is performed along three axes and random add mask is a random crop operation on the training data, where the size of 12 × 12 × 12 data is cropped and replaced by 0. The number of crops is in the range of 0–16 and the location of the crops is random.

### Evaluation metrics

We chose to use Dice similarity coefficient (DSC), mIoU, HD, and ASD to accurately evaluate the segmentation results.

DCS is used to measure the similarity between two sets, and the value range of DSC is between 0 and 1, where 1 indicates complete overlap between sets A and B, and 0 indicates no overlap. In image segmentation tasks, A and B typically represent predicted segmentation results and actual segmentation annotations, respectively.

IoU is the ratio of intersection divided by union, and mIoU is the average of all categories of IoU, which is used to measure the segmentation performance of the model on each category. The higher the value, the better the segmentation performance of the model on different categories.

HD is used to measure the maximum difference between two sets, that is, the maximum value of the shortest distance from a point in one set to another set, reflecting the worst situation between the two sets, that is, the maximum distance between the model segmentation result and the actual annotation. The smaller the value of HD, the closer the two are.

ASD is the average surface distance between two sets, which is the average of the shortest distance from each point in one set to another set. It is used to evaluate the average error of the segmentation boundary, which is the average distance between the model segmentation result and the actual annotation. A smaller ASD indicates a closer segmentation boundary between the two.

### Implementation details

We chose the training framework PyTorch 2.0.0 with AdamW as the optimizer, a fixed learning rate of 5e-4 and a weight decay factor of 1e-5. The network was trained on two Nvidia GeForce RTX 3090Ti GPUs in a Linux environment with the batch size of 2 and 300 epochs.
